# Myopathy in hyperthyroidism as a consequence of rapid reduction of thyroid hormone: A case report

**DOI:** 10.1097/MD.0000000000007591

**Published:** 2017-07-28

**Authors:** Qianrui Li, Yuping Liu, Qianying Zhang, Haoming Tian, Jianwei Li, Sheyu Li

**Affiliations:** Department of Endocrinology and Metabolism, West China Hospital, Sichuan University, Chengdu, China.

**Keywords:** creatine kinase, hyperthyroidism, methimazole, myopathy, propylthiouracil, relative hypothyroidism, thyroid hormone

## Abstract

**Rationale:**

Myalgia and elevated creatine kinase (CK) are occasionally observed during the treatment of hyperthyroid patients. Relative hypothyroidism resulted from rapid thyroid hormone reduction had been promoted as a plausible cause of these myopathic changes, however rarely reported.

**Patient concerns:**

We hereby presented a 20-year-old female with Grave's disease, who developed myopathy and elevated CK during rapid correction of thyroid hormone.

**Diagnoses:**

Relative hypothyroidism-induced myopathy.

**Interventions:**

Antithyroid drug (ATD) dosage was reduced without levothyroxine replacement.

**Outcomes:**

The muscular symptoms were recovered with CK level returned to normal after adoption of the euthyroid status.

**Lessons:**

Differentiation of relative hypothyroidism from other causes of myopathy, especially with the effect of ATD, is important for clinical practice, although difficult in many cases.

## 1 Introduction

Musculoskeletal symptoms and signs are common in patients with thyroid dysfunctions^[1]^ because the skeletal muscle is a major target of thyroid hormone (TH).^[2]^ Hyperthyroidism mainly leads to symptoms like muscle weakness and wasting without creatine kinase (CK) elevation, whereas hypothyroidism causes myalgia and cramping with remarkable CK elevation, suggesting the presence of myopathies.^[1,3,4]^

In recent years, cases of myalgia and CK elevation during the treatment for hyperthyroidism in patients without evidence of hypothyroidism have been reported, and relative hypothyroidism has been raised as an explanation for these myopathic conditions.^[5–11]^ This effect of relative hypothyroidism has been supported by cases of myopathy in patients treated with thyroidectomy and radioactive iodine therapy.^[5,8]^ However, as many reported patients were on antithyroid drugs (ATDs),^[6,7,9–11]^ it was often difficult to exclude the effect of ATDs as the cause of muscular lesions.

We hereby reported a patient with Grave's disease, who developed myalgia and elevated CK when thyroid function normalized rapidly during the treatment.

## 2 Case report

A 20-year-old female presented to our outpatient clinic on March 7, 2014, with intermittent palpitations, hand tremor when making fine movements, weight loss, and hyperdefecation for 2 months. She did not have fatigue, nausea, exophthalmos, ophthalmalgia, or lower limb edema. On examination, she was found with a grade I goiter without palpable nodules. Blood tests noted decreased thyrotropin (TSH, <0.005 mU/L, normal 0.27–4.2), increased free thyroxine (FT4, 63.3 pmol/L, normal 12–22) and free triiodothyronine (FT3, 28.7 pmol/L, normal 3.6–7.5), positive thyrotropin receptor antibody (13.48 U/L, normal <3), and negative thyroid peroxidase antibody and thyroglobulin antibody. Thus, she was diagnosed with hyperthyroidism and Grave's disease, and was put on propylthiouracil (PTU) 100 mg tid plus metoprolol 25 mg bid.

One month later, the aforementioned symptoms improved and her FT4 dropped (33.27 pmol/L). However, the patient complained of new-onset mild fatigue and myalgia. The muscular pain was intermittent and mild, and did not affect the patient's daily activities. Blood tests found slightly elevated aspartate aminotransferase (AST, 53 U/L, normal <35). Hence, PTU was switched to methimazole (MMI) 10 mg tid.

**Figure 1:**
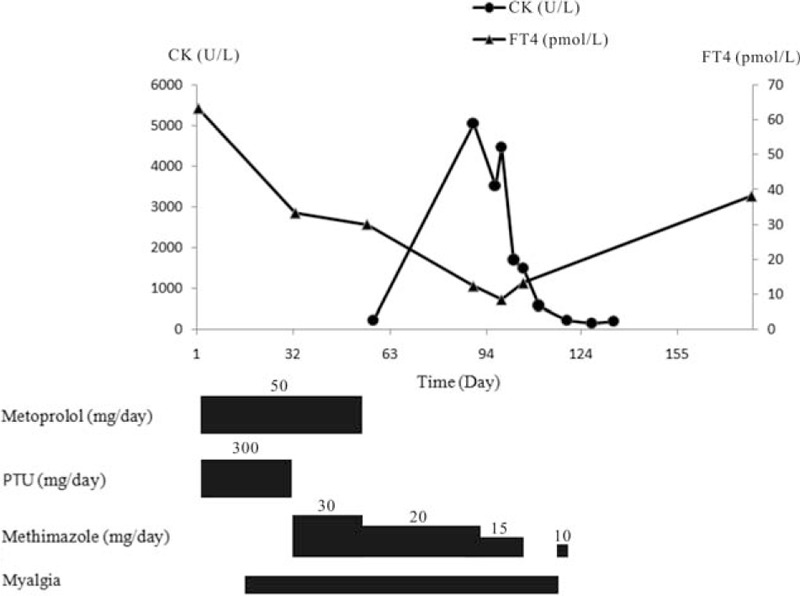
Clinical course of the disease, including prescription, myalgia, and serum levels of thyroxine and creatine kinase. CK = creatine kinase, FT4 = free thyroxine, PTU = propylthiouracil.

Another month later, the patient revisited the clinic with progressed fatigue and myalgia, especially in bilateral shoulders and thighs. The patient had difficulty in combing hair or squatting when the pain occurred. The pain often lasted for around 1 minute and could be relieved with rest. Investigations found elevated CK (228 U/L, normal 20–140) and normal myoglobin. MMI was reduced to 20 mg qd because of the improvement of initial symptoms and the decrease of FT4 to 29.97 pmol/L. The clinical course of this case was shown in Figure 1.

On June 3, 2014, the patient complained of very severe myalgia due to which she could neither comb nor squat. Blood tests found largely dropped FT4 (12.27 pmol/L), highly elevated CK (5058 U/L) and elevated AST (71 U/L), and normal serum potassium (3.86 mmol/L). The antinuclear antibodies (ANA) were negative. The patient was followed up with close monitoring of bloods, and MMI was ceased when FT4 decreased to 8.40 pmol/L. One week after the cessation of MMI, FT4 rose to 13.16 pmol/L, CK declined to 1482 U/L, and the muscular symptoms recovered gradually. Another month later, CK fell to 196 U/L. Radioiodine therapy was given to the patient as a radical cure in September 2014.

Written informed consent was obtained from the patient for this case report.

## 3 Discussion

Myalgia and elevated CK are occasionally observed in patients with hyperthyroidism during the normalization of TH, possible etiologies of which include thyrotoxicosis,^[4]^ thyrotoxic hypokalemic periodic paralysis (THPP),^[12]^ and relative hypothyroidism resulted from rapid TH reduction.^[5,8]^ Adverse effect of ATDs was a widely discussed cause for myopathy in literature,^[13–16]^ but agreement on this effect among clinicians was limited due to insufficient clinical and laboratory evidence. Concurrence of thyroid disorders with neuromuscular diseases can also be an important differential diagnosis.^[17,18]^

In our case, the onset of myalgia and elevation of CK occurred upon rapid reduction of FT4 during the treatment, and was improved without specific treatment or discontinuation of ATDs. In searching for etiology, thyrotoxic myopathy was ruled out based on the nonsynchronous onset of muscular symptoms and thyrotoxicosis. ATDs were also not likely to be causative because MMI was prescribed after the onset myalgia and PTU was ceased weeks before myalgia exacerbation and CK elevation. THPP was ruled out as the serum potassium remained normal during the myopathic period. Given above concerns and noting that CK peaked slightly after FT4 reached its nadir, relative hypothyroidism was considered as an etiology for myopathy in this patient.

Several cases suspected of relative hypothyroidism-induced myopathy have been discussed in recent years; however, the majority did not provide a definite diagnosis.^[5–11]^ The first series of cases of relative hypothyroidism-induced myopathy during the treatment of hyperthyroidism was reported by Suzuki et al.^[9]^ Four adult patients with Grave's diseases developed elevated CK levels with or without myalgia and muscle cramps. These changes occurred 2 to 8 weeks after the initiation of MMI and none of the patients had evidence of a hypothyroid state. Administration of levothyroxine with or without decrease of the dose of MMI was given to the patients and the CK levels of all patients normalized within months. Rapid reduction of TH was considered the cause for these myopathic changes because the onset timing of these changes was closely after the reduction of TH, regardless of treatment options, and the amelioration of these changes happened without discontinuation or decrease of the dose of agents.^[9]^ Later, this myopathy-inducing effect of relative hypothyroidism was supported by reports of patients receiving surgical removal of thyroid or radio iodine therapy.^[5,8]^ Relative hypothyroidism-induced myopathy was also reported in children.^[5–7]^ Although the muscular manifestations and the association between clinical presentations and TH changes in paediatric cases were similar to those in adults, the peak value of CK level was often much higher.^[7]^

Relative hypothyroidism might be overlooked due to the concurrent use of ATDs in many cases. The adverse effects of ATDs was widely discussed, ranging from minor reactions, such as skin rash, to major reactions including agranulocytosis, liver damage, and vasculitis.^[19]^ A set of musculoskeletal symptoms, including myalgia, arthralgia, and arthritis, was described as the “antithyroid arthritis syndrome.”^[20]^ However, none of the currently established adverse events involved muscle injury and CK elevation. Myopathy onset during treatment for hyperthyroidism with ATDs was once considered simply as a rare adverse effect of ATDs.^[13–16,21]^ However, relative hypothyroidism was not carefully excluded in any of these cases. In one recent case attributing myopathy to the effect of carbimazole,^[21]^ the reported patient presented with a FT4 level lower than the normal range at the time when muscular symptoms onset, suggesting an evident hypothyroid state. In another case attributing myopathy to MMI,^[16]^ the reported patient presented with borderline FT4 levels at the lower limit during the 2 weeks when CK level elevated to between 651 and 3775 U/L. Earlier cases simply excluded the effect of rapidly reduced TH based on the nonhypothyroid state defined by thyroid function test.^[13,14]^

Identification of myopathy due to relative hypothyroidism was important for clinical decision-making. However, in daily practice, it was often difficult to fully differentiate between ATD- and relative hypothyroidism-induced myopathies. Sometimes, the adverse effects of ATDs could be clearly discounted, such as one case reported by Mizuno et al,^[7]^ when symptoms improved with continuation of the same ATD dosage while adding levothyroxine replacement. Nevertheless, this definite diagnosis was made only retrospectively with very limited help on treatment strategy during myopathic period.

The mechanisms underlying these myopathic changes remained unclear. The wide distribution of TH receptors in muscular tissue might raise the possibility of myocyte injury and subsequent CK elevation in response to rapid TH reduction, even when the reduced TH was still above the normal range. Hence, when adapted to relative hypothyroid condition, myocytes stopped cracking, and CK went back to normal. In addition, decreased renal clearance of CK and the discrepancies between normalized serum TH level and thyrotoxic skeletal tissue were also possible mechanisms.^[8,9,22]^ Considering very different metabolic effect of thyroid hormone between individuals,^[23–25]^ a genetic component was also suspected because the majority of reported cases were Asians.^[8]^

As for clinical practice, smooth reduction of FT4 might be considered to minimize the risk of myopathy, especially in patients susceptible to hormonal change. For patients with new-onset myopathy during treatment, the in-time adjustment of ATD dosage with or without levothyroxine replacement was critical, especially when relative hypothyroidism was highly suspected.^[7,9,11]^

## 4 Conclusion

In summary, we presented a primary hyperthyroidism patient with severe myalgia and obviously elevated CK due to rapid TH correction, which should not be overlooked, although rarely reported.
